# Prenatal Immune Challenge Differentiates the Effect of Aripiprazole and Risperidone on CD200–CD200R and CX3CL1–CX3CR1 Dyads and Microglial Polarization: A Study in Organotypic Cortical Cultures

**DOI:** 10.3390/life14060721

**Published:** 2024-06-02

**Authors:** Katarzyna Chamera, Katarzyna Curzytek, Kinga Kamińska, Monika Leśkiewicz, Agnieszka Basta-Kaim

**Affiliations:** Laboratory of Immunoendocrinology, Department of Experimental Neuroendocrinology, Maj Institute of Pharmacology, Polish Academy of Sciences, 12 Smętna St., 31-343 Kraków, Poland

**Keywords:** aripiprazole, risperidone, lipopolysaccharide, maternal immune activation, microglia, organotypic cortical cultures

## Abstract

Microglia are the primary innate immune cells of the central nervous system and extensively contribute to brain homeostasis. Dysfunctional or excessive activity of microglia may be associated with several neuropsychiatric disorders, including schizophrenia. Therefore, we examined whether aripiprazole and risperidone could influence the expression of the *Cd200–Cd200r* and *Cx3cl1–Cx3cr1* axes, which are crucial for the regulation of microglial activity and interactions of these cells with neurons. Additionally, we evaluated the impact of these drugs on microglial pro- and anti-inflammatory markers (*Cd40*, *Il-1β*, *Il-6*, *Cebpb*, *Cd206*, *Arg1*, *Il-10* and *Tgf-β*) and cytokine release (IL-6, IL-10). The research was executed in organotypic cortical cultures (OCCs) prepared from the offspring of control rats (control OCCs) or those exposed to maternal immune activation (MIA OCCs), which allows for the exploration of schizophrenia-like disturbances in animals. All experiments were performed under basal conditions and after additional stimulation with lipopolysaccharide (LPS), following the “two-hit” hypothesis of schizophrenia. We found that MIA diminished the mRNA level of *Cd200r* and affected the OCCs’ response to additional LPS exposure in terms of this parameter. LPS downregulated the *Cx3cr1* expression and profoundly changed the mRNA levels of pro- and anti-inflammatory microglial markers in both types of OCCs. Risperidone increased *Cd200* expression in MIA OCCs, while aripiprazole treatment elevated the gene levels of the *Cx3cl1–Cx3cr1* dyad in control OCCs. The antipsychotics limited the LPS-generated increase in the expression of proinflammatory factors (*Il-1β* and *Il-6*) and enhanced the mRNA levels of anti-inflammatory components (*Cd206* and *Tgf-β*) of microglial polarization, mostly in the absence of the MIA procedure. Finally, we observed a more pronounced modulating impact of aripiprazole on the expression of pro- and anti-inflammatory cytokines when compared to risperidone in MIA OCCs. In conclusion, our data suggest that MIA might influence microglial activation and crosstalk of microglial cells with neurons, whereas aripiprazole and risperidone could beneficially affect these changes in OCCs.

## 1. Introduction

Microglia are the primary innate immune cells of the central nervous system (CNS) and are derived from myeloid precursors in the yolk sac [1]. They constitute as much as approximately 10–15% of the total number of CNS cells [2] and stay in close contact and interact with neurons and other types of glia in the brain parenchyma [3]. Under physiological conditions, microglia constantly survey their microenvironment [4] and exert multiple diverse roles, together with the regulation of synaptic activity [5] and neurogenesis [6,7], synaptic pruning [8,9], removal of cell debris [10,11], reorganization of neuronal circuits [12] and trophic support for neurons [3,13]. Foremost, however, as the principal CNS immune effectors, microglial cells represent the first line of defence in response to exogenous threats [14]. Any disturbances or loss of homeostasis triggers a process called microglial activation, characterized by specific morphological changes in these cells and alterations in the expression pattern of various markers, cytokines and chemokines [15,16,17,18].

Literature reports have indicated that dysfunctional or excessive activity of microglia may be associated with several neuropsychiatric disorders, including schizophrenia [19,20]. Postmortem studies have suggested an increase in the activation or density of these cells, inter alia, in the frontal cortices [21,22] and temporal regions [23] of individuals with schizophrenia. Additionally, some meta-analyses and in vivo imaging data have presented similar phenomena in the form of enhanced microglial activation in the grey matter [24] or hippocampus [25] of patients suffering from this condition. Along with these observations, disturbances in the expression and/or protein levels of multiple cytokines (e.g., IL-1β, IL-6, IL-8, IL-10 and TNF-α), chemokines (e.g., CX3CR1) and other factors (e.g., IBA1, TLR4, SERPINA3 and P2RY12), of which microglia are the main reservoir, have been described in relation to schizophrenia [26,27,28,29,30].

The cornerstone in the treatment practice for schizophrenia is medication with antipsychotic drugs [31]. Among these substances, atypical antipsychotics, with two subcategories (second- and third-generation), are currently the most commonly prescribed for this disorder [32,33]. An example of a second-generation antipsychotic is risperidone, which blocks dopamine D_1_ and D_2_ family receptors and affects serotonin (5-HT) transmission with a very high affinity for 5-HT_2A_ receptors. Moreover, this drug displays a moderately high affinity for the H_1_-histamine, α_1_- and α_2_-adrenergic receptors. Unlike other antipsychotics, risperidone does not influence muscarinic transmission [34]. One of the major representatives of third-generation antipsychotics is aripiprazole. This drug acts as a partial agonist of dopamine D_2_, D_3_, D_4_, 5-HT_1A_ and 5-HT_2C_; an inverse agonist of 5-HT_2B_; and an antagonist of 5-HT_2A_ and 5-HT_6_ receptors. Furthermore, aripiprazole binds to α_1_-adrenergic and H_1_-histamine receptors [35].

Multiple findings have indicated that in addition to their neuroleptic activity, antipsychotics modulate inflammatory processes, including microglial activity [36,37]. For instance, Hou et al. [38] showed that olanzapine reduced nitric oxide (NO) release by murine microglial N9 cells subjected to lipopolysaccharide (LPS). Another antipsychotic drug, spiperone, also inhibited the production of NO and attenuated the expression of proinflammatory cytokines (IL-1β and TNF-α) in LPS-stimulated BV-2 microglial cells [39]. Recently, in the same cell line, it was observed that haloperidol, risperidone and aripiprazole changed the proinflammatory action of microglia [40]. In the research by Bian et al. [41], perospirone and quetiapine decreased NO generation and TNF-α levels in IFN-γ-activated microglia in vitro.

Despite the growing evidence of the impact of antipsychotics on microglia, the exact mechanisms underlying these effects are still not fully established. Therefore, due to the implication of these cells in schizophrenia-related pathology, research on this subject is of crucial importance for expanding/improving the available pharmacotherapy. In the present study, we sought to examine whether the selected atypical antipsychotics, specifically aripiprazole and risperidone, could influence the communication between microglial cells and neurons, represented herein by the expression of the *Cd200–Cd200r* and *Cx3cl1–Cx3cr1* axes. Additionally, we evaluated the impact of these antipsychotic drugs on microglial activity in the form of pro- and anti-inflammatory marker expression (*Cd40*, *Il-1β*, *Il-6*, *Cebpb*, *Cd206*, *Arg1*, *Il-10* and *Tgf-β*) and cytokine release (IL-6, IL-10).

Considering that microglial activity is widely affected by interplay with neuronal and other brain-related cells, the present research was conducted using organotypic cortical cultures (OCCs) that maintain functional intercellular and intersystem interactions in ex vivo conditions [42]. To investigate the effects of the tested drugs in the most comprehensive manner, we applied not only OCCs from control rat offspring (control OCCs) but also from animals that were exposed to maternal immune activation (MIA OCCs) with LPS, which is one of the widely implemented approaches to study schizophrenia-resembling disturbances in animals [43,44,45,46]. The experiments were executed under basal conditions and after additional stimulation with the bacterial endotoxin (LPS), according to the “two-hit” hypothesis of schizophrenia [47,48].

## 2. Materials and Methods

### 2.1. Animals

Adult Wistar rats (Charles River, Sulzfeld, Germany) were maintained under standard conditions: room temperature of 23 °C, 12/12 h light/dark cycle (lights on at 6:00 a.m.) and food and water available ad libitum. A group of pregnant females (*n* = 18) was generated as previously described [45,49] and randomly divided into two equal parts: (1) control and (2) MIA. All experimental protocols were approved by the Animal Care Committee of the Maj Institute of Pharmacology, Polish Academy of Sciences, Cracow, and met the criteria of the International Council for Laboratory Animal Science and Guide for the Care and Use of Laboratory Animals (consent numbers: 236/2016 and 128/2018). Every possible effort was made to reduce the number of animals used and minimize their distress and suffering.

### 2.2. Prenatal Exposure to LPS

MIA was generated as previously reported [44,45,49,50,51] by the administration of bacterial endotoxin to pregnant rats. LPS (from *Escherichia coli* 026:B6; Sigma-Aldrich, St. Louis, MO, USA) was dissolved to obtain a concentration of 2 mg/kg of body weight in 1 mL of saline. The solution was subcutaneously administered to females in the MIA group on alternate days starting from the seventh day of pregnancy until delivery, between 9:00 and 10:00 a.m. Control pregnant animals were subjected to the same treatment regimen with the corresponding volume (1 mL/kg) of saline. After delivery, the pups were housed with dams until postnatal days 6–7 (PND6–7). No differences in litter size or weight were observed between the control and MIA offspring.

### 2.3. Organotypic Cortical Cultures

Organotypic cultures were prepared based on the slightly modified procedure of Stoppini et al. [52] from the frontal cortices of animals at PND6–7 from both the control (control OCCs) and MIA (MIA OCCs) groups. The pups were decapitated, and the brains were aseptically removed to be transferred to an ice-cold working buffer consisting of 96% Hanks’ balanced salt solution (HBSS) without salts, 3.5% glucose, 0.4% penicillin and streptomycin solution and HEPES (to maintain the pH) (all from Gibco, Paisley, UK). Subsequently, the frontal cortices were dissected, placed on Teflon discs, transversely cut into 350 μm slices using a McIlwain tissue chopper and situated on ThinCerts^TM^—TC Inserts (Greiner bio-one, Kremsmünster, Austria) with 0.4 μm pore size transparent membranes in 6-well plates. The sections were cultured with 1 mL of Dulbecco’s Modified Eagle Medium (DMEM) + GlutaMax™-I (50%; pH 7.4) enriched with 20.5% HBSS with salts, 25% horse serum (HS), 0.1 mg/mL glucose, 1% amphotericin B, 0.4% penicillin and streptomycin solution, 1% B-27 supplement and HEPES (to maintain pH at 7.4) (all from Gibco, Paisley, UK) in a humidified 5% CO_2_ incubator at 37 °C. After 24 h, half of the medium volume (0.5 mL) was replaced, and then after the next 48 h, 1 mL of medium was replaced with fresh medium. Later, the medium was substituted (1 mL) after 48 h with a new medium containing a reduced amount of HS (10%). On the seventh day of in vitro culture, the medium was replaced with a serum-free 1% N2-supplemented mixture of 50% DMEM F-12 (pH 7.4), 44% HBSS with salts, 0.1 mg/mL glucose, 1% amphotericin B, 0.4% penicillin and streptomycin solution, 1% B-27 and HEPES (to maintain pH at 7.4) (all from Gibco, Paisley, UK).

### 2.4. Chemicals and Drugs

Aripiprazole and risperidone (both from Carbosynth Limited, Berkshire, UK) were reconstituted in dimethyl sulfoxide (BioShop, Burlington, ON, Canada) to 10 mM stock solutions. Aripiprazole was added to the OCCs directly from the stock, whereas risperidone was further diluted before each use in sterile phosphate-buffered saline (PBS; Sigma-Aldrich, St. Louis, MO, USA). The final concentrations in the well were 50 or 100 µM [53,54,55] for aripiprazole and 5 µM for risperidone [56]. LPS from *Escherichia coli* 0111:B4 (Sigma-Aldrich, St. Louis, MO, USA) was dissolved in PBS (Sigma-Aldrich, St. Louis, MO, USA), and the final concentration in the well was 1 μg/mL [57].

### 2.5. Treatment

Thirty minutes following the last medium change, control and MIA OCCs were stimulated with either aripiprazole or risperidone for 2 h and later additionally exposed to LPS for 24 h [57,58,59]. Control groups in both types of OCCs were subjected to the appropriate vehicle in the analogous volume and regimen.

### 2.6. Culture Collection and Sample Preparation

At the end of the treatment, culture media were collected for the measurement of IL-6 and IL-10 protein levels using enzyme-linked immunosorbent assay (ELISA).

Simultaneously, the slices intended for quantitative real-time polymerase chain reaction (qRT-PCR) were mixed with TRI Reagent^®^ (Sigma-Aldrich, St. Louis, MO, USA) and total RNA was extracted utilizing the Chomczynski method [60]. Immediately after extraction, the concentration of RNA was determined by a NanoDrop 1000 Spectrophotometer (ND-1000 UV/Vis; Thermo Fisher Scientific, Waltham, MA, USA).

### 2.7. Quantitative Real-Time Polymerase Chain Reaction

The complementary DNA (cDNA) was synthesised from equal amounts of RNA (0.5 μg) via reverse transcription using an NG dART RT kit (EURx, Gdańsk, Poland). Then, the cDNA was amplified with a FastStart Universal Probe Master (Rox) kit (Roche, Basel, Switzerland) and TaqMan probes (Life Technologies, Carlsbad, CA, USA) for the genes listed in Table 1.

The PCR mixtures consisted of 3 μL of cDNA templates (diluted 10 times in PCR-grade distilled water), 0.5 μL of a specific TaqMan probe, 5 μL of 1× FastStart Universal Probe Master (Rox) mix and PCR-grade distilled water to a total volume of 10 μL. The thermocycling conditions were as follows: 95 °C for 10 min (initial denaturation), 40 cycles at 95 °C for 15 s (denaturation), 60 °C for 1 min (annealing) and 50 °C for 2 min (extension). The threshold value (C_t_) for each sample was set in the exponential phase of PCR, and the ∆∆C_t_ method was used for data analysis.

As the research described in this article was a part of the project evaluating the effects of different antipsychotics (quetiapine, aripiprazole and risperidone) on OCCs under varied conditions, some results (precisely, for the groups: control OCCs + vehicle, control OCCs + LPS, MIA OCCs + vehicle and MIA OCCs + LPS) of qRT-PCR analyses have already been published in the article regarding quetiapine [58]. However, those data are also shown herein due to their importance in presenting the impact of aripiprazole and risperidone on the gene expression of the studied parameters.

### 2.8. Enzyme-Linked Immunosorbent Assay

The protein levels of IL-6 and IL-10 (both from BD Biosciences, San Diego, CA, USA) were measured using commercially available ELISA kits in accordance with the manufacturer’s instructions. The results of ELISA experiments are expressed as the optical density at 450 nm.

### 2.9. Statistical Analysis

Statistical analysis of the data was executed with Statistica 13.0 software (StatSoft, Palo Alto, CA, USA). All biochemical experiments were performed under the same conditions, regardless of the treatment or culture (control OCCs vs. MIA OCCs). The outcomes of all analyses are presented as the mean ± SEM. The normal distribution and the homogeneity of the variance were evaluated with the Shapiro–Wilk test and Levene’s test, respectively. Comparisons of variables between groups were carried out by applying factorial analysis of variance (factorial ANOVA) with Duncan’s post hoc test or planned comparisons via one-way ANOVA (contrast analysis). The results were considered statistically significant when the *p* value was less than 0.05. All graphs were generated in GraphPad Prism 7 software (San Diego, CA, USA).

## 3. Results

### 3.1. Effect of Aripiprazole and Risperidone on Gene Expression of Cd200–Cd200r and Cx3cl1–Cx3cr1 Axes in Control and MIA OCCs under Basal and LPS-Induced Conditions

Microglia are crucial to the adequate development of the CNS and are prime candidates to mediate MIA-induced brain abnormalities [61,62]. The homeostatic properties of these cells come from, among others, the exchange of signals linking them with neurons [63,64]. Simultaneously, the literature reports implicated that antipsychotics may influence the activation of microglia [36]. Therefore, we determined the mRNA levels of two microglial receptors (*Cd200r* and *Cx3cr1*) and their corresponding neuronal ligands (*Cd200* and *Cx3cl1*, respectively) in control and MIA OCCs under basal conditions and after exposure to aripiprazole (50 and 100 µM) or risperidone (5 µM) and/or LPS (Table 2).

As previously presented [58], when quantifying gene expression of the *Cd200–Cd200r* axis, we observed a decreased level of the receptor (*p* = 0.0003) in MIA OCCs when compared to control OCCs (Table 2). Concurrently, MIA OCCs were more susceptible than control OCCs to LPS stimulation in terms of *Cd200r* (*p* = 0.0012) expression. Regarding the impact of antipsychotics on this dyad, we detected lower mRNA levels of the receptor (*p* = 0.0053) in control OCCs after incubation with 50 µM aripiprazole and increased expression of the ligand (*p* = 0.0083) in MIA OCCs subjected to risperidone treatment.

As previously reported [58], exposure to the bacterial endotoxin significantly reduced the mRNA levels of *Cx3cr1* in both control (*p* = 0.0058) and MIA (*p* = 0.0106) OCCs. qRT-PCR analyses revealed that the addition of aripiprazole at a dose of 100 µM to control OCCs resulted in upregulated expression of both *Cx3cr1* (*p* = 0.0054) and *Cx3cl1* (*p* = 0.0229). No changes in the mRNA levels of the *Cx3cl1–Cx3cr1* dyad after interventions with antipsychotics were found in MIA OCCs.

### 3.2. Effect of Aripiprazole and Risperidone on Gene Expression of Microglial Markers in Control and MIA OCCs under Basal and LPS-Induced Conditions

The microglial response to insults to the homeostasis of the CNS generates the release of multiple pro- and anti-inflammatory factors as well as the expression of various CD antigens [65]. Accordingly, we examined the levels of the genes (*Cd40*, *Il-1β*, *Il-6*, *Cebpb*, *Cd206*, *Arg1*, *Il-10* and *Tgf-β*) that are considered crucial for microglial activity in control and MIA OCCs under basal and LPS-stimulated conditions as well as after administration of aripiprazole (50 and 100 µM) or risperidone (5 µM) (Table 3).

As previously described [58], the exposure of control OCCs to the bacterial endotoxin increased the mRNA levels of *Cd40* (*p* = 0.0284), *Il-1β* (*p* < 0.0001), *Il-6* (*p* < 0.0001), *Cebpb* (*p* < 0.0001), *Arg1* (*p* = 0.0003) and *Il-10* (*p* < 0.0001) and lowered *Tgf-β* (*p* = 0.0002) expression (Table 3). Pretreatment of the slices with aripiprazole at both doses applied in the study as well as with risperidone reduced the impact of LPS on *Il-1β* (50 µM aripiprazole: *p* < 0.0001; 100 µM aripiprazole: *p* = 0.0010; risperidone: *p* = 0.0004), *Il-6* (50 µM aripiprazole: *p* < 0.0001; 100 µM aripiprazole: *p* < 0.0001; risperidone: *p* < 0.0001) and *Arg1* (50 µM aripiprazole: *p* = 0.0079; 100 µM aripiprazole: *p* = 0.0030; risperidone: *p* = 0.0121) levels. Simultaneously, in control OCCs subjected to risperidone prior to stimulation with the bacterial endotoxin, the drug upregulated *Cd40* (*p* = 0.0456) expression. Additionally, incubation of control OCCs with aripiprazole at a concentration of 100 µM elevated the mRNA levels of *Cd206* (*p* = 0.0198) and *Tgf-β* (*p* < 0.0001).

In line with previous observations [58], the influence of LPS on MIA OCCs manifested as higher *Il-1β* (*p* < 0.0001), *Il-6* (*p* < 0.0001), *Cebpb* (*p* < 0.0001), *Arg1* (*p* = 0.0002) and *Il-10* (*p* < 0.0001) gene expression and decreased *Tgf-β* (*p* = 0.0001) levels (Table 3). The effect of the bacterial endotoxin on *Il-1β* (*p* = 0.0001) and *Il-6* (*p* = 0.0001) expression in MIA OCCs was less pronounced than that in control OCCs. Notably, the addition of aripiprazole at both doses to MIA OCCs diminished the changes induced by LPS in the mRNA levels of *Il-6* (50 µM aripiprazole: *p* = 0.0005; 100 µM aripiprazole: *p* < 0.0001) and *Arg1* (50 µM aripiprazole: *p* = 0.0145; 100 µM aripiprazole: *p* = 0.0028). Furthermore, aripiprazole at a concentration of 100 µM suppressed the effect of the bacterial endotoxin on *Cd206* (*p* = 0.0150) expression in MIA OCCs. Along with these alterations, the prestimulation of MIA OCCs with 50 µM aripiprazole generated upregulation of the *Cd206* (*p* = 0.0047) level. We did not observe an impact of risperidone on any of the investigated microglial markers in MIA OCCs, and no differences in *Cd40* mRNA expression were found in this type of OCC.

### 3.3. Effect of Aripiprazole and Risperidone on IL-6 and IL-10 Protein Levels in MIA OCCs under Basal and LPS-Induced Conditions

**Figure 1:**
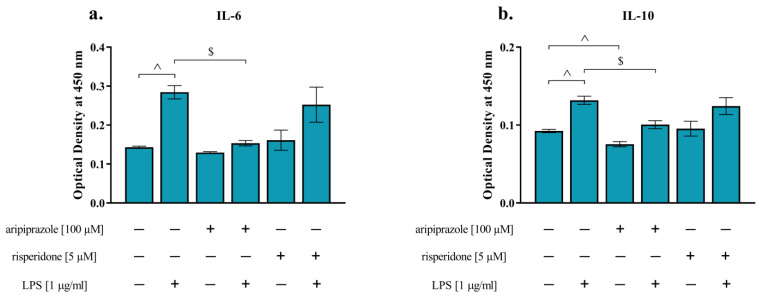
Protein levels of IL-6 (**a**) and IL-10 (**b**) in MIA OCCs after treatment with aripiprazole (100 µM) or risperidone (5 µM) under basal and lipopolysaccharide (LPS)-stimulated conditions. *n* = 4–14. The results were calculated as the optical density at 450 nm and are displayed as the means ± standard errors of the means (SEM). Statistical analysis was performed using planned comparisons via one-way ANOVA (contrast analysis). ^ *p* < 0.05 vs. MIA OCCs + vehicle, $ *p* < 0.05 vs. MIA OCCs + LPS.

In the next set of experiments, we measured the impact of aripiprazole (100 µM) and risperidone (5 µM) treatment on the protein levels of proinflammatory IL-6 and anti-inflammatory IL-10 under basal and LPS-induced conditions in MIA OCCs (Figure 1).

Treatment of the slices with the bacterial endotoxin increased the production of IL-6 (*p* < 0.0001) and IL-10 (*p* < 0.0001). The effect of LPS was diminished by aripiprazole, as the drug lowered the levels of both IL-6 (*p* < 0.0001) and IL-10 (*p* = 0.0005). We did not note any influence of risperidone on the disturbances in cytokine synthesis after stimulation with the bacterial endotoxin. Concurrently, the release of IL-10 (*p* = 0.0487) was decreased by the addition of aripiprazole to MIA OCCs (Figure 1).

## 4. Discussion

Microglial activity is moderated by numerous mechanisms within the brain, including the exchange of signals connecting microglia with other cells, particularly neurons [63]. This communication determines homeostasis, while its malfunctions upregulate the release of proinflammatory factors by and phagocytic activity of microglia [1]. Among these mechanisms, the specialized endogenous protein pairs CD200–CD200R and CX3CL1–CX3CR1 are instrumental and exemplify specific ligand–receptor dyads [64,66,67]. Therefore, the present study evaluated whether aripiprazole and risperidone treatment might modulate MIA-evoked (constituting a model of schizophrenia) changes in the expression of *Cd200–Cd200r* and/or *Cx3cl1–Cx3cr1* axes and microglial polarization in OCCs obtained from neonatal rats subjected either to a prenatal challenge or in a “two-hit” model (represented herein by MIA applied together with additional immune stimulation).

In our research, we observed that MIA diminished *Cd200r* expression and affected the OCCs’ response to additional LPS exposure in terms of this parameter. Simultaneously, the administration of the bacterial endotoxin downregulated the mRNA level of *Cx3cr1* in both types of OCCs. We also demonstrated that risperidone elevated *Cd200* expression in MIA OCCs, while aripiprazole treatment upregulated the gene levels of the *Cx3cl1–Cx3cr1* dyad in control OCCs. Notably, the antipsychotics limited the LPS-generated increase in the expression of proinflammatory factors (*Il-1β* and *Il-6*) and promoted the mRNA levels of anti-inflammatory components (*Cd206* and *Tgf-β*) of microglial polarization, mostly in the absence of the MIA procedure. Finally, we revealed a more pronounced modulating impact of aripiprazole on the expression of pro- and anti-inflammatory cytokines when compared to risperidone in MIA OCCs.

In our earlier studies applying the same experimental scheme of prenatal challenge, we found that the MIA procedure impaired neuron–microglia crosstalk, as depicted by changes in the CD200–CD200R and CX3CL1–CX3CR1 axes in rat brains [45,49]. The anomalies in these ligand–receptor systems were present both in adulthood and at early life stages when crucial neurodevelopmental processes occur [45]. Furthermore, in Sprague–Dawley rats, the majority of MIA-produced alterations were associated with the frontal cortex [49].

Given the abovementioned observations, in the present research, we introduced organotypic cultures prepared from frontal cortices of 6- to 7-day-old control and MIA-exposed offspring. A significant advantage of this approach is the fact that OCCs maintain intact cortical architecture and functional interactions between cells and the neuroimmune and endocrine systems [68,69]. Hence, this model provides exceptional conditions for determining the molecular mechanisms of antipsychotic drug action in the cortex under ex vivo circumstances.

It is well documented that bidirectional communication between neurons and microglia is fundamental for the proper functioning of the brain [70,71]. Cytokines and CDs along with their receptors represent ligand–receptor signalling pathways that are uniquely important for neuron–microglia crosstalk [72,73,74]. Among these molecules, CD200 and CX3CL1 come to the fore due to their cell-type-specific localization. They are principally expressed by neurons when their receptors, CD200R and CX3CR1, respectively, are predominantly present on microglia, resulting in specific axes controlling brain homeostasis [75,76,77]. As we previously suggested, interruptions to this balance contribute to the development of schizophrenia-like disturbances, among others, in the form of behavioural abnormalities [45,49,78,79].

Under physiological conditions, the CX3CL1–CX3CR1 pair governs neurodevelopmental processes, including neuronal survival [80] and synaptic pruning [81]. Therefore, the deficits in *Cx3cr1* expression in MIA OCCs subjected to LPS stimulation, along with the changes in the *Cd200r* level, which we observed in our study, may negatively influence the proper regulation of these processes. Hence, the upregulation of *Cx3cl1–Cx3cr1* expression after aripiprazole administration could be perceived as the normalizing/protective performance of the drug directed on microglia and neurons. Since this beneficial action of aripiprazole was noted solely in control OCCs, we suggest that prenatal challenge determined the response of cortical cells to drug activity. Simultaneously, our present research showed limited consequences of risperidone preincubation, expressed only as upregulation of *Cd200* mRNA levels in MIA OCCs. Nevertheless, this outcome indicates that this antipsychotic also constitutes to some extent a homeostatic effect by targeting neuronal cells. To the best of our knowledge, no previous reports have described an association between the action of aripiprazole or risperidone and the modulation of the abovementioned ligand–receptor axes in OCCs.

Multiple studies have highlighted that CD200–CD200R and CX3CL1–CX3CR1 dyads are neuroinflammatory “off” signals for microglia [82,83]. Dysfunction in these axes exaggerates the proinflammatory response of these cells to immune challenges [84,85] and/or causes a prolonged inflammatory response [86,87]. Microglia-mediated damage in schizophrenia-vulnerable regions, including the frontal cortex and hippocampus, may contribute to further changes in brain structures related to this condition and result directly in cognitive and negative symptoms [21,88,89]. Considering these observations, we examined the mRNA levels of microglial markers and the trajectory of these cells in MIA OCCs after treatment with aripiprazole and risperidone and/or the “second hit” with LPS.

Under basal conditions, aripiprazole upregulated *Cd206* and *Tgf-β* expression in control OCCs. The main impact of this drug was observed after introducing the “second hit”, as aripiprazole inhibited the LPS-induced increase in the expression of proinflammatory cytokines (*Il-1β* and *Il-6*) in control and partially in MIA OCCs. In contrast, a weakening effect of aripiprazole on bacterial endotoxin stimulation in terms of *Arg1* levels might suggest a rather modulatory nature of this antipsychotic’s action on the polarization of microglia in the presence of additional immunostimulants. To date, particular attention has been given to the neurochemical background of aripiprazole pharmacology due to its clinical safety and success in alleviating the positive symptoms of schizophrenia [35,90]. As an atypical antipsychotic, this drug is also effective against the negative symptoms of this disease [91,92] and has been positively validated in various animal models [93,94,95]. In addition to its action on neurotransmitters, aripiprazole exhibited anti-inflammatory characteristics, as it decreased LPS-produced NO release and IFN-γ-induced microglial activation by suppressing Ca^2+^ influx into microglia [96,97]. The inhibitory potential of this drug on microglial activity has also been shown in a poly I:C model, with TRPM7 possibly involved in this effect [98].

Our present results suggest that modulation of the *Cx3cl1–Cx3cr1* axis might be engaged in the anti-inflammatory effect of aripiprazole. Reinforcement of this dyad already under basal conditions may lead to the intensification of favourable polarization of microglia, depicted, among other things, as elevated *Cd206* expression. Recently, CX3CL1–CX3CR1 signalling and the CD206 marker have been highlighted as critical contributors to the regulation of microglial polarization [99]. Simultaneously, in *Cx3cr1*-deficient mice, decreased mRNA levels of *Cd206* were reported [100], which might imply a relationship between microglial CD antigens and receptors crucial for the determination of microglial trajectory.

In contrast to aripiprazole, the anti-inflammatory effect of risperidone was limited and noticeable only in control OCCs subjected to the “second hit”. This antipsychotic reduced LPS-enhanced expression of proinflammatory cytokines (*Il-1β* and *Il-6*) and *Arg1*, and further increased the *Cd40* mRNA level elevated by bacterial endotoxin. Therefore, the balancing influence of risperidone on both pro- and anti-inflammatory factors in OCCs may be postulated. Moreover, the present research indicates an essential role of the MIA procedure, which limits the immunomodulatory response of OCCs to risperidone treatment, and suggests that the rise in *Cd200* expression evoked by this drug was relatively marginal in the mechanism of risperidone action. The lack of a distinct effect of risperidone might also result from the impact of MIA on cultured cells and, consequently, the change in drug uptake and accumulation [101]. Recently, examinations performed in mice revealed that microglia from MIA-exposed offspring had a long-lived reduction (termed by the authors as “blunting”) in the immune response across the developmental trajectory that was accompanied by disturbances, e.g., in chromatin accessibility [102]. Notably, prenatal replacement of these aberrantly formed microglia with naive cells ameliorated the changes in immune reactivity, signifying that MIA severely influences the long-term response of microglia.

In the present study, we found that only aripiprazole inhibited the LPS-induced production of IL-6 and IL-10 in MIA OCCs. This endotoxin is one of the most potent bacterial inducers of cytokine release, including not only proinflammatory (TNF-α, IL-1β and IL-6) but also anti-inflammatory factors such as IL-10 [103,104,105]. LPS triggers the induction of IL-10 secretion, efficiently preventing the expression of proinflammatory factors [106]. Therefore, the impact of aripiprazole seems to be dual-faced. Evidence for the role of aripiprazole and risperidone in regulating cytokine levels in patients with schizophrenia is ambiguous. Noto et al. [107] reported that IL-6, IL-10, TNF-α and IL-4 levels decreased after ten weeks of risperidone treatment. Furthermore, Sobiś et al. [108] found that IL-1β, IL-6 and TNF-α expression declined after four weeks of aripiprazole therapy. Notably, the anti-inflammatory effects of aripiprazole and risperidone were similar across a panel of 21 cytokines except for TNF-α, IL-13, IL-17α and CX3CL1, where aripiprazole appeared to have a more significant effect on this ligand than risperidone [109]. Although these studies were performed on the peripheral blood of patients, they seem to at least in part correlate with our present results. As postulated by Obuchowicz et al. [110], antipsychotics differentially influence the balance between pro- and anti-inflammatory cytokines, depending on glial activation. Under conditions of slight activation, some of these drugs increase IL-10 release without a significant effect on proinflammatory cytokines. However, in high-grade inflammation, they can decrease proinflammatory cytokine levels, often without impacting IL-10 production [110]. Nevertheless, further studies are required to recognize this phenomenon in more detail. The dual impact of aripiprazole on the profiles of both cytokines in our research can point to the involvement of related signalling pathways in its mechanism of action. Paradoxically, the STAT3 pathway can be activated by IL-6 and IL-10 [111]. STAT3 activation in response to IL-6 in combination with anti-inflammatory cytokines (e.g., TGF-β and IL-10) determines the activation of microglial cells [112,113]. Therefore, considering the published data, we postulate that aripiprazole could activate intracellular regulatory mechanisms in MIA OCCs, which in a time-dependent manner might affect STAT3 activation, but this hypothesis requires further studies. The relationship between the inflammatory response in the brain and STAT3 levels was also indirectly observed in our previous research using an in vivo model [114].

## 5. Conclusions

The present study underlines that, in addition to clinical data showing the utility of antipsychotics in the treatment of schizophrenia symptoms, the mechanism of aripiprazole and at least in part risperidone action may be based on the modulation of the inflammatory response and polarization of microglia towards an anti-inflammatory profile. Moreover, our results, for the first time, indicate that specific endogenous neuron–microglia communication systems that are disturbed in pathological conditions (i.e., schizophrenia, neuroinflammation) can be influenced mainly by aripiprazole and can be proposed as a promising new therapeutic approach. Finally, we believe that further data would enhance the significance of the aspects of CD200–CD200R- and CX3CL1–CX3CR1-mediated neuroinflammation and enable the establishment of related signalling pathways for antipsychotic therapy in brain pathology.

## Figures and Tables

**Table 1:** Genes (with corresponding catalogue numbers of TaqMan probes) that were determined in control and MIA OCCs under basal conditions and after exposure to aripiprazole (50 and 100 µM) or risperidone (5 µM) and/or lipopolysaccharide (LPS) using qRT-PCR. *Gapdh* served as the reference gene.

Gene	Catalogue Number
*Cd200*	Rn01646320_m1
*Cd200r*	Rn00576646_m1
*Cx3cl1*	Rn00593186_m1
*Cx3cr1*	Rn00591798_m1
*Cd40*	Rn01423583_m1
*Il-1β*	Rn00580432_m1
*Il-6*	Rn01410330_m1
*Cebpb*	Rn00824635_s1
*Cd206*	Rn01487342_m1
*Arg1*	Rn00691090_m1
*Il-10*	Rn01644839_m1
*Tgf-β*	Rn00572010_m1
*Gapdh*	Rn01775763_g1

**Table 2:** The mRNA expression of *Cd200r*, *Cd200*, *Cx3cr1* and *Cx3cl1* in control and MIA OCCs after treatment with aripiprazole (50 and 100 µM) or risperidone (5 µM) under basal and LPS-stimulated conditions. *n* = 3–7 in control OCCs and *n* = 2–6 in MIA OCCs (*Cd200r*, *Cd200*, *Cx3cl1*), *n* = 3–8 in control OCCs and *n* = 2–6 in MIA OCCs (*Cx3cr1*). The results were calculated as the average fold change and are displayed as the means ± standard errors of the means (SEM). Statistical analysis was performed using factorial ANOVA with Duncan’s post hoc test or planned comparisons via one-way ANOVA (contrast analysis). * *p* < 0.05 vs. control OCCs + vehicle, # *p* < 0.05 vs. control OCCs + LPS, ^ *p* < 0.05 vs. MIA OCCs + vehicle [58].

**Factor**	**Control OCCs**							
	**Vehicle**	**LPS**	**Aripiprazole** **50 μM**	**Aripiprazole** **50 μM + LPS**	**Aripiprazole** **100 μM**	**Aripiprazole** **100 μM + LPS**	**Risperidone**	**Risperidone + LPS**
***Cd200r***	1.04 ± 0.12	0.78 ± 0.08	0.48 ± 0.04 *	0.75 ± 0.15	0.75 ± 0.29	1.11 ± 0.15	0.91 ± 0.13	1.01 ± 0.38
***Cd200***	1.12 ± 0.26	0.80 ± 0.16	0.58 ± 0.16	0.51 ± 0.13	0.57 ± 0.21	0.36 ± 0.15	1.08 ± 0.30	1.20 ± 0.35
***Cx3cr1***	1.11 ± 0.18	0.34 ± 0.08 *	0.97 ± 0.35	0.47 ± 0.05	2.12 ± 0.68 *	0.47 ± 0.08	0.99 ± 0.18	0.55 ± 0.12
***Cx3cl1***	1.03 ± 0.11	1.28 ± 0.21	1.17 ± 0.42	1.01 ± 0.16	2.01 ± 0.47 *	0.92 ± 0.13	0.82 ± 0.23	1.10 ± 0.12
**Factor**	**MIA OCCs**							
	**Vehicle**	**LPS**	**Aripiprazole** **50 μM**	**Aripiprazole** **50 μM + LPS**	**Aripiprazole** **100 μM**	**Aripiprazole** **100 μM + LPS**	**Risperidone**	**Risperidone + LPS**
***Cd200r***	0.33 ± 0.12 *	**0.17 ± 0.02 #**	0.40 ± 0.13	0.33 ± 0.14	0.16 ± 0.03	0.35 ± 0.02	0.55 ± 0.20	0.23 ± 0.04
***Cd200***	0.49 ± 0.13	0.55 ± 0.10	0.75 ± 0.22	0.39 ± 0.09	0.48 ± 0.13	0.30 ± 0.03	**1.62 ± 0.94 ^**	1.07 ± 0.25
***Cx3cr1***	1.08 ± 0.40	**0.29 ± 0.06 ^**	1.03 ± 0.18	0.36 ± 0.05	0.88 ± 0.39	0.24 ± 0.07	1.44 ± 0.25	0.30 ± 0.05
***Cx3cl1***	0.90 ± 0.36	0.51 ± 0.06	0.77 ± 0.13	0.31 ± 0.09	0.60 ± 0.21	0.19 ± 0.03	0.77 ± 0.27	0.70 ± 0.38

**Table 3:** The mRNA expression of *Cd40*, *Il-1β*, *Il-6*, *Cebpb*, *Cd206*, *Arg1*, *Il-10* and *Tgf-β* in control and MIA OCCs after treatment with aripiprazole (50 and 100 µM) or risperidone (5 µM) under basal and LPS-stimulated conditions. *n* = 3–8 in control OCCs and *n* = 2–6 in MIA OCCs (*Cd40*, *Il-1β*, *Il-6*, *Cebpb*, *Cd206*, *Il-10*, *Tgf-β*), *n* = 2–7 in control OCCs and *n* = 2–6 in MIA OCCs (*Arg1*). The results were calculated as the average fold change and are displayed as the means ± standard errors of the means (SEM). Statistical analysis was performed using planned comparisons via one-way ANOVA (contrast analysis). * *p* < 0.05 vs. control OCCs + vehicle, # *p* < 0.05 vs. control OCCs + LPS, ^ *p* < 0.05 vs. MIA OCCs + vehicle, $ *p* < 0.05 vs. MIA OCCs + LPS [58].

**Factor**	**Control OCCs**							
	**Vehicle**	**LPS**	**Aripiprazole** **50 μM**	**Aripiprazole** **50 μM + LPS**	**Aripiprazole** **100 μM**	**Aripiprazole** **100 μM + LPS**	**Risperidone**	**Risperidone + LPS**
***Cd40***	1.06 ± 0.13	1.55 ± 0.21 *	0.84 ± 0.25	1.65 ± 0.33	1.00 ± 0.10	2.00 ± 0.26	1.23 ± 0.21	**2.10 ± 0.24 #**
***Il-1β***	1.05 ± 0.14	119.50 ± 8.15 *	0.39 ± 0.08	**57.16 ± 9.00 #**	0.21 ± 0.06	**80.02 ± 4.85 #**	1.37 ± 0.44	**80.19 ± 11.13 #**
***Il-6***	0.81 ± 0.34	95.49 ± 9.72 *	1.31 ± 0.35	**24.28 ± 8.79 #**	1.29 ± 0.49	**16.10 ± 3.78 #**	0.96 ± 0.30	**42.39 ± 4.36 #**
***Cebpb***	1.03 ± 0.09	2.28 ± 0.23 *	1.11 ± 0.13	2.48 ± 0.19	1.04 ± 0.09	2.29 ± 0.10	0.89 ± 0.11	2.13 ± 0.09
***Cd206***	1.09 ± 0.16	1.02 ± 0.22	1.36 ± 0.19	0.58 ± 0.16	2.35 ± 0.94 *	0.51 ± 0.16	0.76 ± 0.23	0.73 ± 0.11
***Arg1***	1.14 ± 0.26	88.60 ± 23.98 *	1.53 ± 1.04	**19.67 ± 5.55 #**	0.56 ± 0.13	**2.96 ± 0.90 #**	1.99 ± 0.45	**23.74 ± 9.85 #**
***Il-10***	0.13 ± 0.13	35.41 ± 5.69 *	0.11 ± 0.11	33.52 ± 5.91	0.22 ± 0.22	29.40 ± 5.03	0.85 ± 0.21	26.82 ± 4.55
***Tgf-β***	1.06 ± 0.13	0.48 ± 0.05 *	1.18 ± 0.26	0.60 ± 0.04	2.06 ± 0.26 *	0.79 ± 0.04	1.02 ± 0.16	0.61 ± 0.04
**Factor**	**MIA OCCs**							
	**Vehicle**	**LPS**	**Aripiprazole** **50 μM**	**Aripiprazole** **50 μM + LPS**	**Aripiprazole** **100 μM**	**Aripiprazole** **100 μM + LPS**	**Risperidone**	**Risperidone + LPS**
***Cd40***	1.06 ± 0.19	1.14 ± 0.12	0.82 ± 0.08	1.73 ± 0.03	0.62 ± 0.15	1.51 ± 0.21	1.19 ± 0.29	0.94 ± 0.11
***Il-1β***	0.78 ± 0.15	**80.41 ± 9.13 #^**	0.32 ± 0.01	57.41 ± 2.46	0.16 ± 0.01	67.68 ± 5.44	0.67 ± 0.10	92.71 ± 28.94
***Il-6***	1.37 ± 0.21	**60.16 ± 12.60 #^**	2.94 ± 0.84	**13.29 ± 0.66 $**	1.87 ± 0.90	**11.98 ± 0.77 $**	1.21 ± 0.51	58.46 ± 16.87
***Cebpb***	1.02 ± 0.13	**2.28 ± 0.15 ^**	0.62 ± 0.28	1.86 ± 0.37	0.87 ± 0.05	1.80 ± 0.04	0.88 ± 0.17	1.89 ± 0.65
***Cd206***	1.26 ± 0.33	1.85 ± 0.30	**2.87 ± 1.10 ^**	0.72 ± 0.23	1.60 ± 0.56	**0.48 ± 0.16 $**	1.12 ± 0.25	2.02 ± 0.77
***Arg1***	0.71 ± 0.15	**91.96 ± 29.13 ^**	0.80 ± 0.12	**9.83 ± 5.47 $**	0.24 ± 0.05	**3.55 ± 1.40 $**	0.67 ± 0.27	100.11 ± 56.65
***Il-10***	0.49 ± 0.17	**33.06 ± 3.34 ^**	ND	39.94 ± 4.63	ND	34.46 ± 5.95	0.12 ± 0.12	29.39 ± 1.05
***Tgf-β***	1.11 ± 0.17	**0.44 ± 0.03 ^**	1.07 ± 0.11	0.46 ± 0.07	1.11 ± 0.23	0.46 ± 0.07	1.02 ± 0.10	0.40 ± 0.03

## Data Availability

All data supporting the conclusions of this manuscript are provided in the text, figures and tables. Data will be made available upon request.
